# The Role of Positive and Negative Metacognitions About Sex in the Relationship Between Psychological Distress and Problematic Consumption of Pornography. A Mediation Model

**DOI:** 10.1080/19317611.2024.2410943

**Published:** 2024-10-07

**Authors:** Tudor-Daniel Huțul, Adina Karner-Huțuleac, Andrei-Marian Mariș, Iulia-Mariana Filote, Florentina-Brigita Chiricheș, Mădălina Ioana Florentina Mavrichi

**Affiliations:** Faculty of Psychology and Education Sciences, Alexandru Ioan Cuza University of Iași, Romania

**Keywords:** Metacognitions, metacognitions about sex, psychological distress, problematic consumption of pornography

## Abstract

**Objective:**

This study aims to examine how positive and negative metacognitions about sex (MSS) mediate the relationship between psychological distress and problematic consumption of pornography.

**Method:**

821 individuals from Romania, aged 18–70 (*M* = 27.21; SD = 8.74), 64.4% of whom were women.

**Results:**

Both positive and negative MSS mediated the link between psychological distress and problematic consumption of pornography.

**Conclusion:**

MSS plays a crucial role in understanding the dynamics between psychological distress and problematic consumption of pornography. The study contributes to filling a gap in the literature by exploring MSS in this context. Theoretical and practical implications are discussed.

## Introduction

According to the “Diagnostic and Statistical Manual of Mental Disorders, Fifth Edition” (DSM-5), psychological distress encompasses a range of symptoms and experiences that an individual finds troubling, confusing, or beyond the ordinary (American Psychiatric Association, 2013). It is defined in the literature as a distinct feeling of emotional discomfort or disturbance, triggered by a demand or stressor, which leads to temporary or enduring effects on overall well-being (Ridner, 2004). This distress can manifest in various ways, such as anxiety, depression, fear, or feelings of helplessness (Burnette et al., 2020).

When psychological distress arises, individuals often turn to various coping mechanisms to deal with negative outcomes and to manage the challenging situations they face (Algorani & Gupta, 2023; Folkman & Moskowitz, 2004; Huțul et al., 2024). The literature has highlighted that in the absence of other adaptive strategies, pornography can be one of the most accessible coping mechanisms, as individuals with internet access can access it almost instantaneously (Borgogna et al., 2022; Bőthe et al., 2021; Lewczuk et al., 2020). This type of material refers to any sexual content featuring genital organs, whether in the form of pictures or video clips, and created with the intent to sexually arouse the viewer (Kohut et al., 2020). It is well-established that men consume more pornography than women, a phenomenon that holds constant regardless of sexual orientation (Bőthe et al., 2023; Grubbs et al., 2023; Huțul et al., 2024). This can become a coping strategy because it can be used to deal with negative outcomes of mental health such as loneliness, social isolation, psychological distress, or emotional dysregulation (Cardoso et al., 2022; Huțul et al., 2024). Paradoxically, despite its potential to address unpleasant emotional states and, depending on the circumstances, serve as a short-term adaptive strategy, the use of pornography can over time lead to an increase in adverse emotions (Levin et al., 2019), thus being seen as a maladaptive coping strategy (Brand et al., 2016). To better understand this nuance, it is important to underline that Levin et al. (2019) work indicates that the relationship between the frequency of pornography viewing and self-reported negative consequences was mediated by experiential avoidance. This suggests that viewing pornography to avoid distress is associated with increased negative emotion – in the same way that experiential avoidance in many forms is associated with negative emotion – not that pornography itself increases negative emotion.

One of the most common negative outcomes of pornography use is that some individuals may experience psychological distress due to a discrepancy between their behavior and their beliefs (Grubbs et al., 2019). Additionally, a significant reason people experience distress from viewing explicit material is religiosity; religious individuals tend to morally disapprove of pornography, which leads them to suffer as a result of its use (Grubbs et al., 2015, 2018, 2019; Huțul & Karner-Huțuleac, 2023a; MacInnis & Hodson, 2016). In addition to psychological distress due to religious or moral conflict, other situations where pornography may be maladaptive include viewing it in inappropriate contexts, such as at school or work, as well as problems within couple relationships that arise when there are discrepancies between partners in pornography use (Grubbs et al., 2019; Huțul & Karner-Huțuleac, 2024a; Willoughby et al., 2016). However, given that the most important predictor of distress resulting from pornography use is religiosity, it is imperative that any model discussing pornography must also take religiosity into account and at least control for it.

Furthermore, the subject of sexuality and pornography is extremely important in Romania, given the general context in which sexual behavior is highly controversial and regarded as taboo (Gergely, 2023; Huțul, 2023; Karner-Huțuleac & Huțul, 2023). Historically, during the communist period, sexuality was redefined to align with communist ideology (Turcescu & Stan, 2005). The communist ideology in Romania banned any discussion of sexual education. The only aspect that Romanians were to consider was the necessity of having as many children as possible. In this context, on October 1, 1966, Nicolae Ceaușescu implemented Decree No. 770, which prohibited abortions to encourage population growth (Andrei & Branda, 2015; Pop-Eleches, 2010). Additionally, the importation of contraceptives was banned, and local production was reduced to nearly zero, emphasizing the importance of increasing Romania’s population and establishing moral standards that eliminated discussions about sexuality (Pop-Eleches, 2010). Consequently, the suppression of any open discussion about sexuality during the communist regime has led to a lasting negative perception of sexual education and pornography in Romania, which continues to influence societal attitudes today. This situation in the present day is a result of transgenerational pathways, as parents raised during the communist era have passed on their negative perceptions of sexuality and pornography to their children, with the understanding that family values regarding the use of pornography significantly influence children’s attitudes (Huțul & Karner-Huțuleac, 2024a).

As time has progressed, ideas about sexuality have remained consistent, particularly due to religious perspectives – such as what constitutes acceptable and unacceptable sexual behavior in Romania – that have been heavily influenced by the Orthodox Church and political shifts since the collapse of communism (Turcescu & Stan, 2005). This influence is evident in the majority population, based on the statistics showing that in the 2022 census, 13,989,584 people (73.86% of the population) declared themselves of the Orthodox faith (Romanian National Institute of Statistics, 2024). Consequently, Romanian society largely avoids discussions concerning sexuality and its various facets, including sex and/or pornography, reflecting prevailing moral and societal norms (Turcescu & Stan, 2005). In this context, research on pornography use in Romania is almost non-existent. One exception reveals that people are consuming pornography and due to moral disapproval of pornography experience religious distress (Huțul & Karner-Huțuleac, 2023a).

Ultimately, in the Romania context, research on sexual behaviors and pornography is essential. This area has been suppressed and inhibited over the past few decades, despite aspects such as the high level of religiosity, which is extremely prevalent in Romania, potentially leading to disapproval of actions in the sexual sphere, even if they are still pursued, resulting in psychological distress. This psychological distress can, in turn, contribute to the development of psychiatric disorders.

### Meta-cognitions and their role in addictive behaviors

One reason people may engage in ineffective coping mechanisms could stem from their metacognitions, which represents the individual’s ability to recognize their own mental processes and manage them effectively (Flavell, 1979), and extend to the link between cognitive processes and emotional difficulties (Wells & Matthews, 1994). Considering the activities we engage in throughout life that require continuous monitoring of our thoughts and actions, it is rare not to engage in metacognitive processes (Norman et al., 2019). Regarding metacognitive experiences, these refer to the specific perceptions, feelings, and knowledge that an individual has about their own cognition (Efklides, 2008). In the realm of addictive behaviors, metacognitions involve reflections on one’s own thoughts about the effects and consequences of behavior, which can become activated throughout and following involvement in addictive behavior, creating a vicious cycle where these metacognitions influence emotional states that individuals then attempt to manage through continued involvement in the addictive behavior (Caselli et al., 2018; Spada et al., 2015).

On another note, these metacognitions can, in turn, be classified into two categories, positive and negative (Efrati & Spada, 2024). To better understand the difference between positive and negative metacognitions, consider that a positive metacognition might involve a belief such as “drinking alcohol will help me relax and enjoy the party” (Efrati & Spada, 2024) reflecting an expectation about the effects of drinking. In contrast, a negative metacognition could be a thought like “once I start drinking, I won’t be able to stop” (Efrati & Spada, 2024), which reflects a concern about potential loss of control and its consequences.

It is important to note that positive metacognitions encompass beliefs and knowledge related to constructive thinking processes that facilitate the adoption of effective coping strategies (Capobianco et al., 2019; Spada et al., 2015). However, it is crucial to distinguish between positive metacognitions that are genuinely constructive and those that may be based on cognitive distortions. Positive metacognitions can be effective if they lead to healthy coping mechanisms. Yet, if these beliefs are based on distortions, they might contribute to maladaptive behaviors over time, just as the use of pornography may be adaptive in the short term but can increase adverse mental health outcomes in the long term, potentially transforming from an adaptive strategy into a maladaptive one (Brand et al., 2016; Levin et al., 2019). Also, positive metacognitions play an important role because they influence coping strategies and regulatory processes, thereby impacting individuals’ ability to manage addictive behaviors (Capobianco et al., 2019; Spada et al., 2015). For example, a belief like “Thinking about having sex will help me being more peaceful” may initially seem beneficial but could lead to problematic consumption if fosters reliance on pornography as a primary coping strategy when sexual thoughts arise without addressing underlying issues.

Regarding negative metacognitions, they can influence emotion regulation by distorting control efforts, leading to the persistence of negative thinking, which intensifies or prolonging negative emotions (Capobianco et al., 2019). As addictive behavior intensifies, negative metacognitive beliefs about its uncontrollability or frequency may emerge (Spada et al., 2015). For example, research has shown that sexual thoughts can occupy an individual’s mental space to the point of obsession, making it extremely difficult to function in various areas of life and leading to risky sexual behaviors (Efrati & Spada, 2024).

Furthermore, it is important to note that the literature has emphasized the extremely important role of metacognitions in understanding various aspects, such as problematic pornography use (Allen et al., 2017), technology addiction (Casale et al., 2021; Chen et al., 2022), alcohol dependence (Spada & Wells, 2005), smoking addiction (Nikčević & Spada, 2010), or sexual distress (Zarbo et al., 2019).

### Metacognitions about sex and their role in pornography use

Metacognitions about sex may play an important role in the context of pornography use. This situation may arise due to the fact that certain triggers, such as fantasies, memories, and urges, can activate the self-regulatory executive function and are associated with metacognitive beliefs (Efrati & Spada, 2024). Therefore, metacognitions about sex can result from processing intrusions and attempting to suppress thoughts, whether these are referring to positive metacognitions about sex (e.g., “Thinking about having sex will help me being more peaceful”) or negative metacognitions about sex (e.g., “I can’t stop thinking about having sex”) (Efrati & Spada, 2024). To better understand how positive metacognitions about sex and negative metacognitions about sex are conceptualized, the positive ones refer to the use of sexual behavior in managing cognitions, such as “engaging in sex will help me to control my negative thoughts”, and in managing emotions, such as “sexual behavior reduces my anxious feelings”. In contrast, the negative ones focus on uncontrollability, such as “I have no control over my sexual behavior”, and on potential dangers, such as “thoughts about sex interfere with my functioning” (Efrati & Spada, 2024).

As a result of elevated negative emotions and increased sexual urges (Efrati & Spada, 2024), individuals may be more inclined to engage in sexual activities in an attempt to control these sensations and reduce the gap between their desired and actual states. When sexual desires are extremely intense due to metacognitions, whether they are positive or negative, people may turn to pornography to achieve immediate satisfaction (Huțul & Karner-Huțuleac, 2024b) in the absence of other alternatives in real life. This situation occurs because sexual satisfaction can be obtained not only through in-person sexual activities but also through activities such as sexting (Huțul & Karner-Huțuleac, 2022; Parker et al., 2013), reading books with erotic content (Frederick et al., 2017), or even watching pornography (Daspe et al., 2018).

### Putting the pieces together: the potential mediating role of metacognitions about sex

Metacognitions have functioned as a mediator in various studies. For example, they have functioned as a mediator between negative affect, emotion dysregulation, and internet gaming disorder (Marino et al., 2019). Regarding positive metacognitions, while they have a less significant indirect effect, they can trigger emotional distress and serve as a means for cognitive-affective self-regulation (Billieux et al., 2020; Marino & Spada, 2017). Negative metacognitions are more often shown to play a significant indirect role, emerging from underlying beliefs of inadequacy and helplessness, which lead individuals to engage in various potentially addictive behaviors as a form of coping mechanism (King & Delfabbro, 2014; Spada & Caselli, 2017). Additionally, these metacognitions can exacerbate negative emotional states, compelling individuals to persist in their addictive behaviors (Perales et al., 2020; Spada & Caselli, 2017).

Also, in accordance with previous literature (Casale et al., 2016; 2021; Marino et al., 2019), a study conducted by Efrati and Spada (2024) showed that both positive and negative metacognitions about sex mediated the effects of dysregulated thoughts, negative affect, and impulsivity on compulsive sexual behavior. The findings from this study also indicated that individuals experiencing negative emotions might develop obsessive thoughts about sex, which could lead to compulsive sexual behavior. Given that problematic pornography consumption is categorized under the umbrella of impulse control disorders classified as “Compulsive Sexual Behavior Disorder” (CSBD; Stein et al., 2020) in “The Eleventh Edition of International Classification of Diseases” (ICD-11; World Health Organization, 2022), it should not surprise us if metacognitions about sex were to function as a mediator in the relationship between psychological distress and problematic pornography consumption, similar to how the study conducted by Efrati and Spada (2024) showed that negative emotions can lead to compulsive sexual behavior mediated by metacognitions about sex. Our study builds on these findings by examining how metacognitions about sex can mediate the relationship between psychological distress and problematic pornography use. For example, an individual who experiences psychological distress may develop metacognitions like “Engaging in pornography will alleviate my sexual tension” which could lead to increased pornography use as a coping strategy. On the other hand, negative metacognitions, such as “I cannot control my urge to view pornography”, may exacerbate psychological distress and further drive problematic consumption.

We consider that these sex-related metacognitions can act as mediators of the relationship between psychological distress and problematic pornography consumption. When sexual thoughts are used as coping mechanisms, an individual feeling tense or anxious may turn to pornography. In this regard, sex-related metacognitions can influence how one responds to sex and can represent a space where this psychological distress is managed. Additionally, referring to positive metacognitions, if an individual believes that engaging in sexual activities reduces their sexual tension, they may be more inclined to seek pornography when experiencing stress. Furthermore, when sexual thoughts are intense, and the individual feels psychological distress due to their inability to fulfill their own sexual desires, problematic pornography consumption may result through sex-related metacognitions.

**Figure 1.**
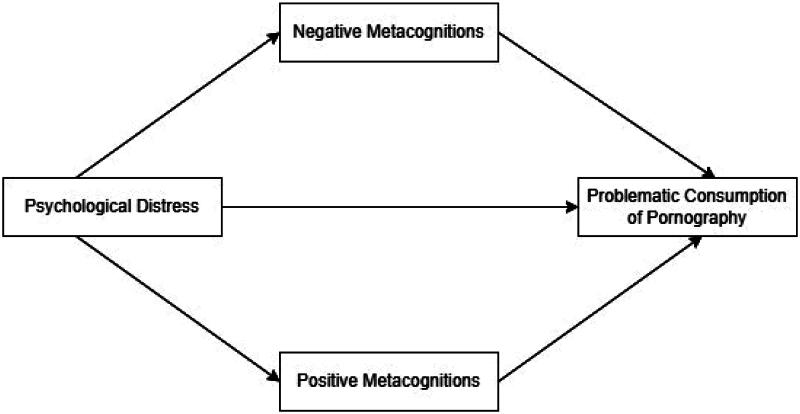
Proposed conceptual model.

Taking into account the aforementioned aspects, the overall conceptual framework for our study is presented in Figure 1.

### The present study

The main objective of the current paper is to advance knowledge by discussing metacognitions about sex in the context of the direct relationship between psychological distress and problematic pornography consumption. From our knowledge, this is the first study in the literature to aim at filling this important gap regarding the role of metacognitions about sex in psychological distress and problematic pornography consumption.

We aimed to explore this direct relationship because, despite the dual nature of the relationship between psychological distress and problematic pornography consumption, individuals may resort to pornography as a coping mechanism when faced with negative mental health outcomes such as psychological distress, potentially leading to the development of problematic pornography consumption. Also, exploring the role of metacognitions about sex in this context is important as it can provide relevant insights that can be subsequently utilized, including in clinical settings by clinical psychologists, psychotherapists, and other mental health workers dealing with problematic pornography consumption.

To explore the potential mediating effect of metacognitions about sex, both negative and positive, in the relationship between psychological distress and problematic pornography consumption, we formulated the following hypotheses.

## Hypotheses

**H1:** Psychological distress predicts problematic consumption of pornography.

**H2:** Negative and positive metacognitions about sex mediate the relationship between psychological distress and problematic consumption of pornography.

## Method

### Participants and procedure

The sample in this study consisted of 821 participants, who were not compensated in any way for their involvement in this research. Their participation was entirely voluntary. The mean age was 27.21 years (*SD* = 8,74), ranging from 18 to 70 years old. The main characteristics of the individuals involved in our study can be observed in Table 1.

**Table 1.** Descriptive statistics for participants.

	*N*	%
Sex		
Male	287	35
Female	529	64.4
Non-binary	5	0.6
Residence		
Urban area	539	65.7
Rural area	282	34.3
Studies		
Pre-universitary	111	13.5
University in progress	300	36.5
Post-universitary	410	49.9
Personal income		
<2000 RON/month	207	25.2
2000–5000 RON/month	288	35.1
>5000 RON/month	219	26.7
I don’t want to answer	107	13
Marital status		
No relationship	317	38.6
In a relationship	336	40.9
Married	143	17.4
Divorced	21	2.6
Widowed	4	0.5
Religion		
Orthodox	524	63.8
Catholic	84	10.2
Protestant	18	2.2
Atheist or agnostic	184	22.4
Other	11	1.3
I don’t want to answer	0	0
Total	821	100

Regarding the procedure, the battery of instruments was created in Google Forms and distributed in general groups on social media platforms such as Facebook and Reddit. Also, a control question was included at the beginning of our Google Form to obtain informed consent from the participants. They were required to read the consent information and then check a box to proceed to the next section. The chosen groups were not specifically targeted (e.g., groups of students from various universities were avoided), but rather selected from the most general ones possible (e.g., groups of residents of Iași city, groups of residents of Vaslui city, groups of residents of Suceava city, etc.). Thus, obtaining responses strictly from a particular specific category of participants was avoided. Additionally, at the end of completing the battery of instruments, participants were shown a message thanking them for their involvement in this research. Alongside this message of gratitude, there was also a note inviting volunteers to share the questionnaire with their acquaintances if they found the research interesting. Thus, a snowball sampling technique was also employed. The data collection period was between February 15, 2024, and April 1, 2024.

The study involved a single inclusion criterion, namely being at least 18 years old at the time of completing the set of questionnaires. Also, prior to filling out our battery of instruments, all participants were asked to read the informed consent, where they received all information regarding data protection and the option to withdraw from the study at any time, as well as the mention that participation is 100% voluntary and they will not be compensated for their participation. In addition, the contact number of the corresponding author was provided in case they had any additional questions regarding the present work. The average filling time was 10–15 minutes.

The study was approved by the Ethics Committee of the Faculty where all authors are affiliated (No. 241/09.02.2024).

### Measures

The tools were translated from English into Romanian using the Backward method. Recommendations for translation and adaptation of the scales were made according to protocol (Beaton et al., 2000; Hambleton & Zenisky, 2010; Maneesriwongul & Dixon, 2004; Sousa & Rojjanasrirat, 2011).

Thus, to translate the instruments into Romanian, two teams of psychologists were formed. All psychologists involved have expertise in studying the variables involved. Each team independently translated the measures, resulting in two translation versions. These two versions were then compared, and through discussions, a final consensus was reached to create a final version of the instruments. After this step, an official translator with expertise in psychology conducted a back-translation of the ultimate version of each scale into English. Once again, two translation versions resulted: the one produced by psychologists and the one produced by the authorized translator. These versions were again compared and discussed to arrive at a single final translation variant. Through this method we applied, we guarantee the retention of the intellectual meaning of the original measures.

In accordance with recommendations from the literature, prior to completing the instruments, the following definition of pornography was provided: “Pornography refers to any sexually explicit films, video clips or pictures displaying the genital area, which intends to sexually arouse the viewer; this may be seen on the internet, in a magazine, in a book or on television.” (Kohut et al., 2020). This definition has also been provided in other studies involving the Romanian population (Huțul & Karner-Huțuleac, 2023b, 2024a), demonstrating that it accurately conveys the original meaning as offered in the literature (Kohut et al., 2020).

#### Metacognitions about sex

To assess this construct, we employed the “Metacognitions About Sex Scale” (MSS; Efrati & Spada, 2024). This instrument contains 11 items grouped into two subscales, measuring positive metacognitions (e.g. “Engaging in sex distracts my mind from problems”) about sex and negative metacognitions about sex (e.g., “I have no control over my thoughts about sex”). The responses are rated on a four-point Likert scale (1 = completely disagree; 4 = completely agree), and the total scores for each dimension (ranging from 11 to 44) are summed and a higher score indicates a greater level of metacognitions about sex (positive or negative). The internal consistency of the MSS in the present study was excellent for both dimensions: *Positive Metacognitions* (*α* = 0.90); *Negative Metacognitions* (*α* = 0.90).

#### Psychological distress

To assess the level of psychological distress, we utilized “The Depression, Anxiety, and Stress Scale” (DASS-21; Lovibond & Lovibond, 1995). This approach to measure psychological distress has been used in numerous other studies, including some that employed the Romanian population (Foti et al., 2023; Măirean et al., 2023). The scale consists in 21 items (e.g., “I found it hard to wind down”) rated on a four-point Likert scale (0 = not apply to me at all; 3 = applied to me very much or most of the time). The scores (ranging from 0 to 63) are summed and as the score gets higher, the higher the psychological distress gets. The internal consistency of the DASS-21 in the present study was excellent (*α* = 0.94).

#### Problematic consumption of pornography

To assess problematic pornography use, we utilize the “Problematic Pornography Consumption Scale” (PPCS; Bőthe et al., 2018). This instrument consists in 18 items (e.g., “I felt that pornography was an important part of my life”) rated on a seven-point Likert scale (1 = never; 7 = every time). The scores (ranging from 18 to 126) are summed and a higher score indicates a greater level of problematic pornography use. This instrument has demonstrated its psychometric properties inclusively on samples consisting of Romanians (Huțul et al., 2024). In the present study, the internal consistency was excellent (*α* = 0.95).

#### Frequency of pornography use

To assess the frequency with which individuals view pornography, we utilized a single item (“How often have you voluntarily viewed pornographic material in the last 6 months?”), which has also been used in other works (Huțul & Karner-Huțuleac, 2023a). The participants had to rate on a seven-point Likert scale (1 = only once in the last 6 months; 7 = daily or almost daily). Given the importance of this construct in the literature regarding pornography consumption, we have decided to control for this variable in the present study. Higher scores indicate a greater frequency of pornography use.

#### Religiosity

To assess religiosity, following other studies on the topic of sexual topics (Grubbs et al., 2020), we utilized 3 items (e.g., “I consider myself religious”) rated on a seven-point Likert scale (1 = strongly disagree and 7 = strongly agree). Responses were averaged. An Alpha Cronbach of *α* = 0.92 was obtained. Similar to the case of frequency of pornography use, given the importance attributed to religiosity in the literature concerning pornography use, we have decided to control for this variable in the present work. Higher scores indicated a higher level of religiosity among respondents.

#### Socio-demographic data

Participants were asked to report their age, sex, place of origin, level of education, relationship status, financial situation, and religious orientation.

### Overview of the statistical analysis

Firstly, we conducted preliminary analysis, and then we tested the associations between the main variables of the study – psychological distress, negative metacognitions about sex, positive metacognitions about sex, problematic consumption of pornography, age, relationship duration, religiosity, and frequency of pornography use. We used an independent samples *t*-test to determine whether there are significant sex differences in problematic consumption of pornography. We conducted regression analysis to examine the relationship between psychological distress and problematic consumption of pornography. Finally, we tested the mediating role of metacognitions about sex (positive and negative) on the relationship between psychological distress and problematic consumption of pornography, using the SPSS macro PROCESS – Model 4, with a 95% confidence interval (CI) and 5000 bootstrapped samples.

## Results

### Preliminary data analyses

We computed the Skewness and Kurtosis values to assess the normality of the distributions (Table 2). All the Skewness values were within the 2/-2 limit and Kurtosis values were within 7/-7, indicating normality, as suggested by Hair et al. (2010) and Byrne (2013). Statistical analyses were performed using the SPSS program, Version 26 (George & Mallery, 2019).

**Table 2.** Descriptive statistic and associations among the main variables.

	M (SD)	Skewness (SE)	Kurtosis (SE)	1	2	3	4	5	6	7	8
1. Psychological distress	19.36 (14.96)	0.72 (0.08)	−0.24 (0.17)	–							
2. Positive metacognitions about sex	8.53 (3.76)	0.36 (0.08)	−0.94 (0.17)	0.07*	–						
3. Negative metacognitions about sex	1.94 (0.13)	1.49 (0.08)	1.46 (0.17)	0.27**	0.44**	–					
4. Problematic consumption of pornography	30.96 (18.91)	1.92 (0.08)	3.3 (0.17)	0.22**	0.38**	0.65**	–				
5. Age	27.21 (8.74)	1.57 (0.08)	2.31 (0.17)	−0.26**	−0.01	−0.02	−0.02	–			
6. Relationship duration (months)	39.13 (79.50)	6.34 (0.08)	7.06 (0.17)	−0.12**	0.02	0.004	0.005	0.43**	–		
7. Religiosity	9.90 (5.85)	0.35 (0.08)	−1.14 (0.17)	0.06	−0.15**	−0.01	−0.09**	−0.01	−0.03	–	
8. Frequency of pornography use	3.07 (2.18)	0.69 (0.08)	−0.79 (0.17)	0.03	0.30**	0.38**	0.58**	−0.06	−0.03	−0.31**	–

*Note*: *p<.05; **p<.001.

### The association between main variables

The results of the correlational analysis (Table 2) suggest that there are significant positive associations between psychological distress, negative metacognitions about sex, positive metacognitions about sex, and problematic consumption of pornography.

### Sex differences regarding the problematic consumption of pornography

The results of the independent samples *t*-test suggest that there are sex differences in problematic consumption of pornography (*t*_(398.04)_ = 11.02; *p* < .001). On average, men (*M* = 41.27) scored higher in problematic consumption of pornography compared to women (*M* = 25.20).

### Testing the regression

The results of the linear regression analysis indicate that psychological distress significantly predicts problematic consumption of pornography (*R =* 0.22; *R^2^ =* 0.05; $R_{\text{adj}}^{2}$ *=* 0.049; *p* < .001; *F_(1; 827)_* = 43.31). Psychological distress is a significant and positive predictor of problematic consumption of pornography (*B =* 0.28; *SE =* 0.04; *β =* 0.22; *p* < .001). Although psychological distress is a significant predictor, the effect size is quite small.

### Testing the mediating role of negative and positive metacognitions about sex on the relationship between psychological distress and problematic consumption of pornography

**Figure 2.**
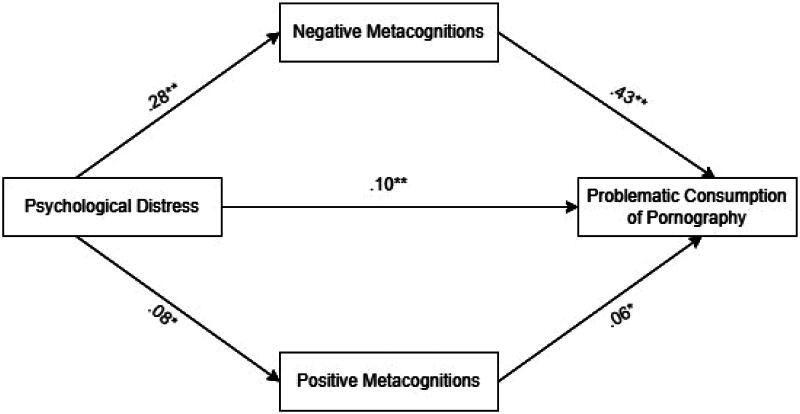
The mediating role of negative and positive metacognitions about sex on the relationship between psychological distress and problematic consumption of pornography. *Note*: *p<.05; **p<.001; the coefficients presented are standardized coefficients

We explore the potential mediating roles of the negative and positive metacognitions about sex on the link between psychological distress and problematic consumption of pornography, controlling for sex, age, religiosity, and frequency of use. The results suggested (Figure 2) that the total effect of psychological distress and problematic consumption of pornography was significant. The direct effect was significant. Both negative metacognitions about sex, and positive metacognitions about sex, had a significant indirect effect on the relationship between psychological distress and problematic consumption of pornography. Although the indirect effects were significant, the effect sizes – particularly for positive metacognitions – were quite small.

## Discussions

The present study has revealed a series of highly important aspects regarding problematic pornography consumption and the role that metacognitions about sex play and how they can influence it. Firstly, our findings have revealed that psychological distress is correlated with problematic pornography use. Our result is consistent with the literature, which has shown that when individuals experience negative outcomes related to mental health, such as loneliness, social isolation, emotional dysregulation, or psychological distress they can turn to pornography use as a coping strategy (Cardoso et al., 2022; Huțul et al., 2024). The reason individuals may resort to using pornography as a coping strategy is its near-instantaneous accessibility for anyone with internet access (Borgogna et al., 2022; Bőthe et al., 2021; Lewczuk et al., 2020). However, while it can sometimes serve as an adaptive coping mechanism in the short term, its use can over time lead to adverse emotions (Levin et al., 2019), thereby evolving into a maladaptive coping strategy in the long term (Brand et al., 2016). This situation, in which pornography use gradually leads to adverse emotions, arises in particular contexts; for instance, Levin et al. (2019) study demonstrates that the link between the frequency of pornography consumption and self-reported negative outcomes was mediated by experiential avoidance. This implies that using pornography as a means to avoid distress correlates with heightened negative emotions – in the same way that experiential avoidance in various forms is linked to negative emotions – rather than pornography itself being the direct cause of increased negative emotions. It is important to emphasize that our findings indicate a correlation rather than a causal relationship. The observed association does not imply a causal relationship, nor does it rule out the possibility of a third variable influencing both phenomena. Although, another potential explanation for this result is that, in this context, the use of pornography, as a maladaptive coping strategy, prevents individuals from healthily managing difficult situations. By not accessing adaptive alternatives, a vicious cycle is reinforced, where they watch even more pornography to regulate their negative states. Consequently, this can lead to problematic consumption. Moreover, our result is in line with the literature which has shown that ineffective coping mechanisms can stem from metacognitions and individuals’ inability to recognize their own mental processes and manage them efficiently (Flavell, 1979).

On another note, our results have indicated that the relationship between psychological distress and problematic consumption of pornography was mediated by positive metacognitions about sex. Our findings suggest that individuals with positive metacognitions about sex may be more susceptible to resorting to pornography when encountering psychological difficulties, thereby reinforcing problematic pornography consumption. Nevertheless, while our findings confirm a statistically significant mediation effect, the magnitude of this effect appears relatively small. This suggests that positive metacognitions about sex may contribute only modestly to the relationship between psychological distress and problematic pornography consumption.

It is important to consider that when an individual has a positive metacognitions about sex, such as “Thinking about having sex will help me being more peaceful” (Efrati & Spada, 2024), might reflect cognitive distortions or errors in thinking. These metacognitions may lead individuals to believe that any sexual activity, including pornography consumption, could serve as an immediate remedy for negative emotional states. Thus, positive metacognitions about sex can serve as a means for cognitive-affective self-regulation (Billieux et al., 2020; Marino & Spada, 2017). Additionally, they might also create a mental framework where pornography is perceived as a quick fix for sexual needs, similar to activities such as sexting (Huțul & Karner-Huțuleac, 2022; Parker et al., 2013) or reading erotic books (Frederick et al., 2017). Moreover, past literature has shown this in the case of pornography use (Daspe et al., 2018). Therefore, while the mediation effect of positive metacognitions about sex is statistically significant, it is crucial to acknowledge that these metacognitions might involve cognitive distortions that could contribute to problematic consumption.

Also, our findings have indicated that the relationship between psychological distress and problematic consumption of pornography was mediated by negative metacognitions. One argument for this result is a negative metacognitions such as “I can’t stop thinking about having sex” (Efrati & Spada, 2024), can intensify a cycle of obsessive thinking. This negative metacognition about sex may reflect an intense and persistent concern regarding the potential uncontrollability of sexual thoughts, which can exacerbate psychological distress in the individual. Considering that this situation can bring about more psychological distress, individuals may resort again to an accessible coping strategy, such as pornography use (Borgogna et al., 2022; Bőthe et al., 2021; Lewczuk et al., 2020).

An important aspect to note is that negative metacognitions about sex, compared to positive metacognitions about sex, were a stronger mediator in the relationship between psychological distress and problematic consumption of pornography. This result can be explained by several factors. For example, negative metacognitions about sex such as “I can’t stop thinking about sex” or “Sexual thoughts control me” can intensify psychological distress because they reflect a nature of thoughts that can create a perceived lack of control and a sense of threat regarding one’s own thoughts, given that the lack of control and fear regarding the danger of thoughts are related to negative metacognitions (Darnell et al., 2024). In this way, psychological distress can be high, and individuals may resort to quick and accessible coping mechanisms such as pornography for rapid temporary escapism (Borgogna et al., 2022; Bőthe et al., 2021; Lewczuk et al., 2020). Another explanation could be that negative metacognitions about can lead to obsessive rumination (Hamonniere et al., 2020), which in turn intensifies distress and generates a greater need for coping, which can be met by pornography. Likewise, our result can be attributed to the fact that positive metacognitions about sex might promote the use of pornography in a more acceptable manner, whereas negative metacognitions about sex can perpetuate intrusive and compulsive thoughts.

In conclusion, our study has shown that negative and positive metacognitions can play an important role in influencing outcomes of a behavior with addictive potential, in accordance with previous literature (Casale et al., 2016, 2021; Efrati & Spada, 2024; Marino et al., 2019). Also, our study aligns with the literature that has shown that metacognition is an important factor in understanding aspects such as technology addictions (Casale et al., 2021; Chen et al., 2022), alcohol dependence (Spada & Wells, 2005), smoking addictions (Nikčević & Spada, 2010), or sexual distress (Zarbo et al., 2019). At the same time, our results also suggest that beliefs about sex, as reflected in metacognitions, may be a negligible predictor (for positive metacognitions) to moderate predictor (for negative metacognitions) of behavior, surpassing other factors. This observation aligns with research indicating that beliefs can significantly influence behavior, underscoring their pivotal role in understanding and predicting behavioral outcomes.

## Limitations and future directions

In analyzing the findings presented in this study, it is essential to address a series of significant limitations that researchers should consider in the future to enhance these results. Firstly, we need to discuss the fact that the instruments we used are self-report type, and these involve a high degree of subjectivity from individuals, which could lead to potential recall bias, as well as to involve socially desirable responses from participants (Adams et al., 1999). Thus, future studies could also focus on more objective measures, such as a concrete assessment of the time spent viewing pornography (for example, the average number of hours participants spend viewing explicit content per week) or the perspective of life partners regarding individuals’ pornography consumption. Secondly, although a method previously used in other studies was employed to attempt to reach a more diversified respondent base, it was done so through social media platforms, and individuals who engage with these platforms may not be representative of the broader population. Subsequent studies should involve alternative data collection methods, especially for individuals who are not acquainted with social media. In this regard, snowball sampling technique may exacerbate this participant selection bias, as those who recommend others to participate likely share similar characteristics, thereby rendering the sample homogeneous. At the same time, the current study did not include an optimal method to prevent receiving responses from bots or false participants, which future research should consider implementing. Thirdly, the cross-sectional design utilized constrains the ability to establish causality between variables. Future studies may employ longitudinal or experimental designs to provide more robust evidence for causal relationships among our constructs, namely psychological distress, metacognition about sex, and problematic pornography consumption. Fourthly, we must address the limitation that while the mediating effects of positive metacognitions about sex are statistically significant, the coefficients are quite small. It is possible that the statistical significance of positive metacognitions about sex may be an artifact of a large sample size rather than theoretically meaningful. Therefore, future studies should take this aspect into consideration.

At the same time, given the importance of moral incongruence in the literature regarding pornography consumption, another limitation of this study is that moral incongruence was not measured. From a moral incongruence perspective, we can argue that metacognitions could mediate the relationship between pornography consumption as the independent variable and psychological distress as the dependent variable: the moral disapproval of pornography consumption and using pornography leading to psychological distress. For example, the negative metacognition “I have no control over my thoughts about sex” may lead to more distress, whereas someone may experience less distress about pornography consumption if they believe “Engaging in sex distracts my mind from my problems”. Future studies should consider this aspect and also measure the concept of moral incongruence, with the present work focusing on the relationship where psychological distress leads to pornography consumption, this relationship being a dual one.

Finally, it is important to note that this research involved measures related to metacognitions about sex in general, as well as a specific measure focusing on pornography use. Thus, the results might have varied if there had been a closer alignment between the metacognitive measures and the outcome variable specific to pornography. Therefore, we wish to emphasize a potential research direction that involves examining both positive and negative metacognitions specifically within the context of pornography use. In this regard, we provide potential examples of such metacognitions (Table 3). Building on these examples, future studies could further explore metacognitions about pornography consumption and consider developing a scale to measure these positive and negative metacognitions with greater specificity.

**Table 3.** Examples of metacognitions regarding pornography consumption.

Positive metacognitions	Negative metacognitions
Pornography consumption may offer me new ideas to enhance my sexual life.	Pornography consumption makes me feel guilty about myself.
Pornography consumption will help me relieve sexual tension and improve my mood.	Pornography consumption can affect my body image, perception of sex, or my intimate relationships.
Pornography consumption can provide me with relaxation during times of high stress or anxiety.	Pornography consumption may diminish my confidence in my ability to maintain a healthy relationship.
Pornography consumption may stimulate my sexual desire and help me connect with my own desires.	Pornography consumption can reduce my interest or pleasure in real sexual activities.
Pornography consumption can enhance self-awareness and help me better understand my own sexuality.	Pornography consumption can lead me to social isolation.

### Theoretical and practical implications

From a theoretical perspective, the present work contributes to enhancing knowledge regarding the constructs of psychological distress, problematic pornography consumption, and metacognitions about sex. Specifically, revealing the mediating roles of both positive and negative metacognitions about sex in the relationship between psychological distress and problematic pornography use adds to the existing literature.

From a practical standpoint, our results may have implications for clinical psychologists, psychotherapists, and other mental health workers. They can incorporate the theme of metacognitions about sex into their practice with individuals experiencing problematic pornography use, now supported by theoretical evidence in this regard. Therapists can assist individuals experiencing problematic pornography use by helping them identify and understand their own metacognitions related to sex through discussions about their thoughts and beliefs regarding sex and pornography consumption. For example, if an individual’s negative metacognitions about sex influence their pornography consumption, therapeutic strategies might include techniques aimed at developing a greater sense of control and reducing obsessive rumination. At the same time, individuals with problematic pornography use can benefit from psychoeducation regarding how both negative and positive metacognitions can influence their behaviors and emotions. They should be informed through psychoeducation about how their thoughts concerning sex and pornography consumption impact their behavior, and how altering these thoughts can aid in reducing problematic pornography use. Additionally, non-governmental organizations and individuals advocating for healthy attitudes toward sexuality and psychoeducation regarding sexual behaviors can also utilize our findings to substantiate their interventions. Furthermore, another practical implication of our study is represented by the potential development of an assessment and intervention guide based on metacognitions about sex. Creating such a guide or protocol for intervening in problematic pornography consumption based on metacognitions about sex could enhance clinical practice.

## Data Availability

All data associated with the article are available upon request to the corresponding author.
